# Selective, reciprocal and quiet: lessons from rural queer empowerment in community-supported agriculture

**DOI:** 10.1007/s10460-024-10552-9

**Published:** 2024-02-16

**Authors:** Guilherme Raj

**Affiliations:** Copernicus Institute of Sustainable Development, Utrecht University, Utrecht, The Netherlands

**Keywords:** Grassroots initiatives, Community power, Gender, Sexuality, Sustainable food systems

## Abstract

Rural queer studies, viewed through the lens of relational agriculture, offer critiques of heteropatriarchal norms in farming and highlight strategies used by queer farmers to manoeuvre discrimination and thrive in rural areas. This paper responds to recent calls for further scrutiny of the experiences of gender and sexually underrepresented groups in community-supported agriculture (CSA). It investigates the empowerment of rural queer people in CSA Guadiana, South Portugal, through the experiences of 12 queer members. I collected data through participant observation, semi-structured interviews and a focus group and analysed them through open coding, followed by focused coding. Results indicate that CSA Guadiana, despite not originally designed for this purpose, facilitates various forms of empowerment and active engagement among queer members, particularly influenced by the leadership of queer producers and recurrent gatherings in queer-owned farmland. Three key lessons of queer empowerment in CSA Guadiana emerge from the analysis and contribute to debates on the politics of recognition, queer community action and visibility in the rural context: (i) self-confidence to perform queerness may be restricted to a selective rural community; (ii) partnerships between producers and co-producers may enable reciprocal queer empowerment; and (iii) queer leadership in agri-food community action may quietly represent gender and sexual diversity in the countryside. These findings offer the rural queer literature novel insights into the complexities, contradictions and limitations of empowerment experienced by queer farmers, artisanal food producers and consumers in a rural CSA.

## Introduction

“Often, [male senior neighbouring farmers] offer us a hand because they want to ‘help the girls’. For them, we must make do because we are two women, and there isn’t a man responsible for the farm. They see us like ‘poor little ones. They don’t have a man to get away with, so they need someone to help’.” (Ana).

Ana is a queer^1^ artisanal food producer and goatherder living with her partner on their farm in rural Alentejo, South Portugal. Ana is one of the founding members of community-supported agriculture Guadiana (GUA). GUA is a collectively organised CSA where members (producers and co-producers^2^) share accountability for various CSA operations such as food production, distribution, community building and decision-making. Co-producers pre-finance a harvest season through a six-month contract, securing the producers’ income and receiving a weekly share of the harvest. In addition to the six-month contract, co-producers can purchase directly from a curated list of local artisanal producers specialised in cheese, bread, nuts, jam and fruits sourced from farms ranging from 1.7 to 3.4 hectares. The findings suggest that rural queer people^3^, like Ana, experience their queerness with greater dignity when participating in CSA compared to their interactions with other local agri-food actors unrelated to the CSA. Such contrasting experiences reveal heteropatriarchal discrimination^4^ at the foundation of the agri-food system in rural Alentejo. However, it remains uncertain whether and how GUA, an initiative that focused on agri-food collaboration and not on gender and sexuality activism, influenced its queer members’ experiences with gender, sexuality and agriculture in rural Alentejo.

Currently, the queer population in Portugal benefits from a legal framework that ensures equal rights in different segments of society, as well as protection against discrimination and hate crimes; yet, such achievements are a work in progress, and gender and sexuality inequalities remain engrained in social structures and everyday life in the country (Esteves et al. 2021; Santos 2022). Five decades of right-wing dictatorship (1926–1974), 80 years of the criminalisation of homosexuality (1912–1982), and prevailing Catholic and heteronuclear family values are historical legacies that hinder further progress in social change towards gender and sexuality diversity and inclusivity (Santos 2022). Notwithstanding these barriers, queer people in GUA experience empowerment in spite of the prevailing heteropatriarchal social order in rural Alentejo.

In this study, I investigate whether and how queer members of GUA feel empowered to become active and thriving members of the CSA. I contribute to research on rural queerness that has discussed the participation of queer farmers in CSA yet calls for further scrutiny of the struggles and achievements of gender and sexually underrepresented groups in this agri-food provisioning scheme (Leslie 2017). Research on rural queerness has examined structural and everyday factors that shape the pursuit of flourishing queer livelihoods in the countryside. In doing so, prior research has contributed to a heterogeneous view of sexual and gender diversity in rural life (Gorman-Murray et al. 2013; Johnson et al. 2016); challenged the “metronormative” bias in LGBT movements and scholarship that has mainly focused on the lives of urban queers (Halberstam 2005); and unveiled the constraints caused by the family farm institution on queer farmers (Hoffelmeyer 2020). To examine queer empowerment in CSA, I draw on the notion of “relational agriculture” (Leslie et al. 2019), which sheds light on “the often-hidden ways that gender and sexual relations organize food production on all farms, calling for gender and sexuality to be understood as central to the study of food systems, rather than a niche topic” (p. 867).

Thus far, the literature on CSA has overlooked the intersection between sexuality and agriculture, let alone the extent to which CSA is a viable model to counter heteropatriarchy. Much remains to be explored. Does CSA offer the means for queer members to pursue their envisioned agri-food system and livelihoods in the countryside and if so, how? What are the possible manifestations of queer empowerment in CSA, including its contradictions and limitations? How do different dimensions of this agri-food provisioning model enable queer empowerment, and how do they differ from other forms of community action? To address this gap, this paper builds upon studies on gender relations in CSA that provide a conceptual lens to approach CSA as a political space where gendered concerns about agri-food practices, norms and structures are expressed and where emancipatory strategies, particularly for women, are lived through everyday politics (Delind and Ferguson 1999). These studies view empowerment in agriculture in relation to gender, thus offering an analytical framework consistent with “relational agriculture” (Leslie et al. 2019).

I conducted participant observation, interviews and focus group involving 12 queer and three cis-gender heterosexual members of GUA. This study finds that while GUA was not originally designed to empower marginalised gender and sexual groups in rural Alentejo, the leadership of queer producers and their recurrent gatherings in queer-owned farmland proved vital for queer empowerment and active engagement in the collective. GUA provided a supportive environment for queer members to collaborate with both queer and cis-hetero^5^ people while confidently expressing their queerness. Conversely, they faced heteropatriarchal discrimination when interacting with cis-hetero local agri-food actors unaffiliated with GUA. Remarkably, queer empowerment within GUA was limited to a socio-economically privileged group in rural Alentejo and constrained by the absence of internal discussions on gender and sexuality.

## Literature review

### Exploring queer lives in the countryside

Rural queer studies have offered critiques of the heteropatriarchal organisation of rural communities, including rural agri-food systems, that pose restrictions to queer flourishing in the countryside^6^. Against a monolithic understanding of queer lives in rural agri-food systems and rural communities more broadly, these hindering factors affect queer people differently across gender, ethnicity/race, class and other social markers of difference (Leslie et al. 2019). For example, the experience of discrimination can vary for a white ciswoman, a Latina ciswoman, and a lesbian Latinx due to their unique social positioning (Hoffelmeyer 2021). In terms of everyday heteropatriarchal discrimination, studies have highlighted experiences of oppression, discrimination, silencing and hiding lived by gender and sexually underrepresented groups in rural communities (Gorman-Murray et al. 2013; Johnson et al. 2016). Within rural agri-food systems, studies have reported queer farmers’ experiences of outright harassment or microaggressions. Microaggressions are “brief, daily assaults on minority individuals, which can be social or environmental, verbal or nonverbal, as well as intentional or unintentional” (Balsam et al. 2011, p. 163, as quoted in Leslie 2017). Examples include verbal harassment and violent body language from neighbouring farmers, intimidating gazes in conventional food venues and probing questions about relationship status by co-workers (Hoffelmeyer 2021; Leslie 2017). Queer farmers may feel constrained to address microaggressions; for instance, queer farmers in CSA who rely on bringing volunteers and customers to their farms may not confront heterosexist remarks to avoid economic risks (Leslie 2017).

At a structural level, the imaginaries of rural communities, access to farmland and the family farm institution are interwoven with heteropatriarchy. Cultural imaginaries of rurality often depict rural communities as exclusionary, lacking in sexual and gender diversity and dangerous for queer individuals (Gorman-Murray et al. 2013; Johnson et al. 2016). Although farmland is affordable, the perception of rural spaces as heterosexist discourages queer people from moving to the countryside (Leslie 2019). Moreover, queer farmers, particularly trans and cis-gendered women, struggle to access farmland (Leslie 2019; Wypler 2019). Queer farmers have been denied private and public credit to purchase or manage land because credit institutions grant credibility to farm units based on heteronuclear relationships that combine romantic and work partnerships (Hoffelmeyer 2021; Leslie 2019; Wypler 2019). Notably, the solidification of the family farm institution in agri-food systems restricts recognition and valorisation for queer farmers that may deviate from the conventional combinations of professional and private lives in the organisation of farm work and living space (Hoffelmeyer 2021).

Despite these difficulties, rural queer studies foreground strategies developed by queer people to manoeuvre heteropatriarchy and enact and protect their agency to pursue desired careers and lifestyles in the countryside. Particularly relevant for this study are the analyses of queer farmers’ community action that create queer spaces in rural areas through formal and informal networks. Queer farmers’ networks are social and physical spaces that strive to minimize biases, criticisms and threats and where queer people build personal connections, exchange farming resources or knowledge and establish collective support and collaborations (Leslie 2019; Wypler 2019). These networks offer participants an opportunity to enjoy a farming space deviant from the predominant heterosexual family farm environment and to imagine and embody alternatives to agrarian heteronormativity (Leslie 2019; Wypler 2019). Outside queer networks, queer farmers navigate the politics of rural recognition and visibility to ensure acceptance in rural communities. They may enact the “sameness” tactic to downplay their queerness and assert other normative identity traces such as asserting themselves to be “just another farmer” to ensure social and commercial ties (Hoffelmeyer 2021). Similarly, queer farmers may disclose their queerness only to those they trust or find relevant to be upfront about their identities, such as to find employment in queer-inclusive farms or bond with other rural queer farmers or customers (Hoffelmeyer 2021).

### Exploring gender relations in CSA

In this study, I draw lessons from women’s empowerment in CSA to investigate the experiences of queer members. This approach is valuable because research on gender relations in CSA sheds light on particular dimensions of these initiatives that can potentially empower participants in the face of patriarchy, sexism and related forms of oppression. While the connection between queer and feminist theories is debated (Williams 1997), I align with authors who address the theoretical limitations of both bodies of work and seek to foster a dialogue between them (e.g., Showden 2012; Marinucci 2016; Andrucki 2021).

Studies on gender relations in CSA claim that CSA initiatives are not catalysers of fundamental political, economic or gender-based reform in agri-food systems and society. Yet, through the relationships of everyday life and the continuous negotiation and implementation of common practices and solutions in CSA, women create visibility for gender issues and assert personal and work relations consistent with their worldviews and objectives (Delind and Ferguson 1999; Jarosz 2011). These studies have taken the standpoint of women farmers and consumers to understand participation and resource management in CSA and referred to empowerment when reporting women’s emancipatory strategies, self-determination and self-confidence experienced in CSA.

I distinguish three dimensions of CSA discussed in the literature on gender relations in CSA that may contribute to queer empowerment. First, CSA creates community relationships through which members perform a “quiet form of activism”: proactive and conscious individual and personal acts to create relationships with food, the environment and people that reflect a lifestyle consistent with their values (Delind and Ferguson 1999). For instance, women farmers feel empowered to establish community relationships based on their desired work-life balance (Jarosz 2011). Second, CSA offers a farmer–consumer partnership to negotiate the costs and terms of distribution and farm operations (Cone and Myhre 2000). This partnership can empower women entering agriculture, enabling them to experiment with diverse farming methods, create alternative mechanisms for sharing risks, ensure equal access to agriculture knowledge and reduce the gender income gap in agriculture (Fremstad and Paul 2020). Third, CSA creates a horizontal organisation that allows farmers and consumers to negotiate and work through day-to-day issues and practical solutions for agri-food operations (Cone and Myhre 2000; Delind and Ferguson 1999). Through this everyday politics, the personal becomes political, and women’s desires, worldviews and intentions shape how CSA re-creates and perpetuates smaller-scale, people-focused, nature-friendly and community-based agriculture (Jarosz 2011; Wells and Gradwell 2001).

## Analytical framework and methods

### Empowerment framework

I adopt the framework of empowerment developed by Allen (2021) that results from a thorough review of feminist approaches to empowerment. Empowerment is conceptualised as a “capacity or ability, specifically the capacity to empower or transform oneself and others” (Allen 2021, p. 18). Allen’s framework has been applied to analyses of power relations in grassroots initiatives (Ahlborg 2017; Raj et al., 2022, Raj et al., 2023). It provides a multifaceted typology of power and empowerment that enables a fine-grained analysis of different yet interrelated real-life manifestations of power affecting the struggles and achievements of grassroots initiatives such as CSA.

In the remainder of this section, I introduce the conceptual framework for studying queer empowerment in CSA based on Allen’s typology of empowerment (Table 1, see section “Four types of queer empowerment”). I focus the analysis and discussion on the first four types of empowerment, as they are highly relevant to the case study. In contrast, manifestations of power feminism were not identified in relation to queer empowerment in GUA.7 Power feminism refers to the intentional individual choice to exercise power over others (Allen 2021). It is consistent with an individualistic, self-assertive, aggressive manifestation of the will to power, in opposition to the notion of women’s victimisation. In the case of GUA, queer participants shared personal stories in which they self-asserted their queerness without caring for others’ opinions or confronting oppressors; however, none of these stories were related to the CSA, nor could I draw connections between those stories and their participation in the CSA. I view the four types of empowerment as not mutually exclusive but as reciprocal possibilities and overlapping experiences influenced by the historical and situated context of those empowered. Moreover, such an empowerment typology opposes an understanding of this term as power-over, often linked to acts of domination and control embedded in oppression and subjection (Allen 2021). However, I contend that empowerment is not an all-encompassing experience. Instead, it complies with the ambivalent and intersectional nature of emancipatory processes that imply contradictory and limiting effects; for example, women farmers may comply with and resist various aspects of subordination in agriculture (Jarosz 2011).

The first two columns of Table 1 show how I operationalised Allen’s (2021) typology for the case of queer empowerment in CSA. The last two columns refer to the empirical findings which, in turn, are organised by the type of empowerment and how they intersected with each of the three dimensions of GUA. The dimension “producer–co-producer partnership” is an adaptation of the “farmer-consumer partnership” term used in the literature on gender relations in CSA. I chose this adaptation as it aligns with the terminology used by the members of GUA, as I explain in more detail next.

## Methods

### Case study: CSA Guadiana

I adopted a single case study approach. For several reasons, CSA Guadiana, located in rural Alentejo, South Portugal, was a relevant case for documenting and analysing queer empowerment in rural agri-food systems because, to start, it was a suitable case to address the call for research on rural queerness in initiatives pursuing alternatives to industrial agriculture (Leslie 2017). GUA was part of the Portuguese CSA Network and shared the network’s common goal of promoting food sovereignty, food as a commons and agroecology. GUA was one of the few active agri-food initiatives that envisioned an alternative to rural Alentejo’s dominant industrial agri-food system. Historically, this region has offered the main stage for modernising the Portuguese agri-food sector (Calvário 2022). Presently, it remains predominantly characterised by large-scale monoculture and greenhouse farms mainly producing olives, berries and other commodities for export (INE, 2021). However, this industrial agriculture model relies on the exploitation of immigrant workers attracted by perceived advantages within national legal frameworks, despite facing precarious labour and living conditions (Pereira et al. 2021).

GUA also linked farmers, artisanal food producers and consumers in rural Alentejo and offered a dynamic and contrasting socio-cultural context to investigate rural queer empowerment. The demographics of rural Alentejo are simultaneously marked by low population density, population decline, an ageing population (INE, 2022) and an increasing neo-rural^8^ population. Neo-rurals are mainly immigrants from Brazil (INE, 2022) but also from other European urban centres seeking a lifestyle change (Esteves 2017; Novikova 2021). The findings suggest that the empowerment and agency of queer members in GUA were contingent on the region’s socio-cultural context. Participants perceived a notable contrast between the progressive views of the neo-rural participants and the conservative local culture, characterised by prominent heterosexual social norms, traditional gender roles and lack of queer spaces. This observation echoes previous research highlighting the cultural shock and mutual estrangement between neo-rural people and the native population in rural Alentejo (Esteves 2017).

Queer and cis-hetero neo-rural farmers founded GUA in the summer of 2019. GUA has never positioned itself as a queer-inclusive CSA and queer members primarily discovered the collective through word-of-mouth and personal connections with the founding farmers. I distinguish among two general types of members in the CSA, as identified by the CSA members. Producers are the horticulture farmers and food producers who manage and execute farm activities. Co-producers are the local consumers who pre-finance the costs of a harvest season, receive fresh produce weekly and can participate in decision-making and work activities organised by the CSA. I refer to co-producers and producers of GUA together as members. In 2019, the CSA counted 10 members; during the fieldwork, that number oscillated between 17 and 24. The fluctuation in membership occurred as producers and co-producers entered or exited the CSA during the renewal of the six-month contract.

### Data collection

I visited GUA for the first time in April 2021. With that visit, I meant to introduce my work and propose a collaboration for another study. However, I was excited to see how freely queer partners shared affection in the group and how gender seemed fluid and not a fixed category shaping roles on the farm and in the collective. It was the first time I had not felt the need to filter my sexuality and gender in a CSA and, more broadly, in the countryside. I felt self-confident and thrilled to self-affirm my queerness that day and throughout the fieldwork campaign. The embodied experience within a queer community actively engaged in agri-food operations has sparked my curiosity to explore how queer members of GUA felt within this space, particularly regarding their experiences of empowerment. I collected data through desk research and fieldwork from April 2021 until May 2022. I carried out participant observation in different formats and at distinct moments throughout the year. To start, I volunteered at the horticulture farm of GUA for three consecutive weeks in July 2021, which allowed me to follow everyday CSA operations and observe the power relations between members. I also participated in the weekly CSA gatherings to assemble and distribute fresh produce, CSA assemblies and other social events and celebrations organised by the group. These moments allowed me to follow their everyday experiences inside and outside the CSA and enriched my understanding of whether and how CSA affected their lives and how their lives, in turn, have affected the CSA.

My sample was composed of 15 members of GUA who self-identified as queer (*n* = 12) or cis-gendered heterosexual (*n* = 3). Queer members’ sexualities were self-identified as bi-sexual (*n* = 4), gay (*n* = 3), fluid (*n* = 3), trans fluid (*n* = 1) and undefined (*n* = 1), and their gender as cis-women (*n* = 7), cis-men (*n* = 3), creative (*n* = 1) and non-binary (*n* = 1). All participants ages ranged from 20 to 55, and most were between 30 and 45 years old. Queer participants were mainly international, originating from Brazil (*n* = 3), Spain (*n* = 2), Germany (*n* = 2), Italy (*n* = 1), the Netherlands (*n* = 1) and Morocco (*n* = 1), and only two were from Portugal, of which only one was born and raised in rural Alentejo. Cis-hetero participants were migrants from other areas of Portugal (*n* = 1) and Germany (*n* = 2). Participants’ occupations covered diverse areas of interest, such as farming, chef, filmmaking, and journalism. Regarding ethnicity, the sample was mainly White (*n* = 6) and European Mediterranean^9^ (*n* = 5), but also multiracial (*n* = 2), African Mediterranean (*n* = 1) and Latin Jewish (*n* = 1).

I selected participants through snowball sampling. I interviewed queer (*n* = 10) and cis-hetero (*n* = 2) CSA members. All participants mentioned in this study provided informed consent before the start of the interview and were given a copy of the audio file and transcription of the interview. I conducted interviews in Portuguese–my first language–or English as an alternative for those who did not speak Portuguese. Topics covered in the semi-structured interviews included the types of heteropatriarchal discrimination encountered in the CSA or more broadly in the region; experiences of and opinions about being an LGBTQIA + member of the CSA; the barriers and opportunities for getting involved in the CSA; and the visions for sustainable agriculture. Additionally, I organised a focus group with CSA members (*n* = 9), of which six were queer people who also participated in the semi-structured interviews, with two additional queer and one cis-hetero members. During the focus groups, participants discussed their understanding of (dis)empowerment of queer people, the values and principles of GUA and the advantages and disadvantages of creating a queer-inclusive community in GUA. To ensure confidentiality, I followed Leslie (2017) and assigned each participant a pseudonym based on the most common names currently used in their country of origin, as stated by governmental agencies. For instance, I used the list of most common names in Brazil published by the Brazilian government’s news agency to choose the pseudonyms for participants originally from Brazil (Agência 2021). To ensure the anonymity of the CSA initiative, I assigned it the fictitious name “Guadiana”, the name of an important river in Alentejo.

### Data analysis

I analysed data through open coding and then focused coding (Benaquisto and Given 2008). I codified the interviews and focus groups with the NVivo qualitative data analysis software. I used open coding to identify emergent themes in and across the interviews and the focus group. I then used focused coding to categorise the data according to the analytical framework’s empowerment typology and CSA dimensions (Table 1). While a literature review informed the identification and choice of the three dimensions of CSA, their relevance for this study emerged from the empirical investigation of the specific case study. Open coding helped me to identify empowerment themes obscured in the literature review or that I had not previously seen, and focused coding allowed me to draw connections between these emergent themes and the analytical framework. In the final stage, I used the analytical framework to identify and compare experiences of empowerment reported by queer CSA members or observed during participant observation. The analysis of empowerment offered insights into which elements of GUA helped queer members feel empowered, including contradictions and limitations, thus suggesting initial understandings of how queer people found the means, through CSA, to overcome or manoeuvre heteropatriarchal discrimination in rural Alentejo and empower themselves to be active and thriving members of the collective.

## Results

First, I present the results as per each dimension of CSA: community relationship, producer–co-producer partnership, and horizontal organisation. I introduce the different types of empowerment reported by queer members of GUA and highlight their contradicting and limiting effects. Remarkably, queer members reported heteropatriarchal discrimination only in relation to actors not engaged with GUA, so “outside” the CSA. Then, I synthesise the types of empowerment identified and then analyse how they are interconnected and affect one another in and across three dimensions of GUA.

### Queer empowerment related to different dimensions of CSA Guadiana

#### Community relationships: “When queer people like me enter the CSA, we are not creating anything new. We are just another queer person.”

The following results highlight the experiences of queer people within the context of community relationships fostered by GUA. Specifically, I explore three key aspects within the reported empowering experiences: heightened self-confidence to express queerness in the group, the assertion of control over queer identities during agri-food transactions and confidence to expand gender and sexuality expressions despite prevailing heteronormativity in rural Alentejo. Producers and co-producers of GUA gathered once a week to distribute fresh produce from the different CSA producers at the farm owned by Ana, a 39-year-old fluid cis-woman producer who sold cheese and pastry at the GUA, and her partner Antônia, a 44-year-old fluid cis-woman who sold bread for the CSA. Interviewees stressed that the recurrence of community gatherings at queer-owned farmland created a social, physical and cultural space in rural Alentejo that alleviated sexual and gender discrimination and where queer people felt safe expressing their queerness. Remarkably, all queer CSA members interviewed said it felt “natural” to be queer in the CSA. The stories of co-producers Miguel and Matteo illustrate how this feeling of naturality enabled queer members to experience heightened *self-confidence that deactivated internalised oppression* repressing their queerness. Miguel, a 40-year-old gay cis-man who worked as a chef at a local hotel venue, experienced microaggressions in the workplace. There, he felt exposed to unpleasant, macho and invasive comments made by his manager and primarily identified as a homosexual by co-workers, which made him constantly self-aware of his sexuality at work.

I am self-aware of my sexuality, like in the hotel where I work. I work only with other Portuguese male cooks from this region. It is all right, and I can be who I am when I am there. However, for them, my sexuality is an essential characteristic of my personality. While in the CSA, I do not feel the same way. I do not even remember that I am gay.

Similarly, Matteo, a 50-something-year-old gay cis-man, felt natural being gay in the CSA. Matteo referred to a generational legacy that imbued him with a “filtering mindset” upon which he (un)consciously decided which personality traces, including sexuality, were (in)appropriate to show to others. Matteo commented, “there was no need for this filter mindset in the CSA because we are just ourselves.”

Furthermore, queer members *took control over their queer selves* and chose when and how to express queerness when purchasing and selling food in GUA. The experiences of co-producers Laura and Valeria, both 32-year-old bi-sexual cis-women, showed the contrast between expressing queerness in GUA and other agri-food venues in rural Alentejo, where they felt more exposed to discrimination. Laura attended the weekly CSA gatherings with her female partner and felt comfortable being open about their relationship, “Most people knew that we were together, and I *definitely* talked about her as ‘my girlfriend’”. In contrast, Laura experienced overt harassment when shopping for groceries with her partner at the local farmers’ market:

A male heterosexual farmer unexpectedly started asking about my relationship with my girlfriend quite provocatively. I honestly answered, “She is my girlfriend.” And then he was like, “Oh, but why? It would be better if you were friends” [shows frustration in her voice]. That was so random. I was buying from him. Why would you harass your client? Anyway, I put down my stuff and did not buy from him in the end.

Likewise, Valeria and her female partner experienced (c)overt harassment at local cafés. They felt targeted by intimidating gazes from other customers, whom they observed were often senior male Portuguese locals. Once, Valeria confronted a senior male Portuguese customer and heard, in return, that two women together were “a perversion, a vice”. Conversely, when referring to her experience in the CSA, Valeria spoke of implicit safety and celebrated the role played by Ana and Antônia to help discard the need to protect or declare her queer identity:

As the leading producers of the CSA, Ana and Antônia are setting an example by showing their homosexuality very naturally and not hiding it. When queer people like me enter the CSA, we are not creating anything new. We are just another queer person.

According to queer members of GUA, they felt inspired by the community relationships to *expand their identities and gain confidence to explore thriving sexual expressions*. In contrast, queer members viewed rural Alentejo as homogeneous, regarding heterosexuality and traditional gender roles, and lacking queer spaces.

The stories of Valeria, Laura, Adilah and Maria illustrate such an empowering experience in GUA. Each of their individual experiences highlighted how sexuality, rurality and agriculture intersected with other social markers of difference in their identity formation. First, Valeria’s case highlighted the intersection of sexuality, rurality and gender. Valeria felt empowered by the encounter with other queer people in the CSA to reaffirm her queerness, despite hostile and sexist experiences in rural Alentejo.

This region is hostile. Like the machismo and the type of masculine models in the region. This aggression and this treatment of women are horrible. It pushed me to escape heterosexuality. So, the homosexual path became more relevant to me. The CSA was essential because it showed that, within the hostility of this region, it is possible to explore diverse sexual orientations. So yes, the CSA might have empowered me to explore my homosexuality further.

Second, Laura’s case illustrated the intersection between sexuality and neo-rurality. Laura, born and raised in a European capital, found in the CSA a community of people who motivated her to pursue a farming career while continuing to explore her sexuality. After leaving her partner, Laura felt encouraged by the group of rural queers in the CSA to stay in the countryside. Third, in the case of Adilah, sexuality and nationality shaped her queer identity formation through the CSA. Adilah, a 37-year-old Moroccan bisexual cis-woman, experienced a heightened sense of freedom in the CSA to explore both prefigurative agri-food practices and queerness, mainly because the CSA gathered a group of international members seeking a lifestyle change: “When we leave our hometown, we feel freer to do what we want, to be who we want” (Adilah). Last, Maria’s case showcased the intersection between sexuality and age. Maria, a 50-something-year-old trans-fluid whose gender was asserted as creative,^10^ saw in the CSA an opportunity to unlock shyness or fear related to their sexuality. Maria called the fear of revealing and exploring sexuality a “restricted conditioning” inherited from a generational legacy.

In sum, the distribution operations of GUA required recurrent gatherings, hence fostering community relationships among members. The gatherings’ location–queer-owned farmland–and the leading role played by queer producers helped create a safe space that alleviated gender and sexual discrimination and strengthened social ties and trust among all CSA members in ways that queer people felt self-confident about their queerness and released internalised oppression. Queer co-producers took control over their queer selves and were less exposed to discrimination in GUA than in other agri-food venues in rural Alentejo. In the context of rural Alentejo, GUA offered a safe social, physical and cultural space for queer people to expand their identities and explore thriving sexual expressions.

### Producer–co-producer partnership: “CSA members trust and appreciate my work. When selling outside the CSA, […] my cheese production is viewed only as a hobby.”

The following paragraphs delve into the key empowering experiences reported by queer members in relation to the producer—co-producer partnership established in GUA. I emphasise three distinct aspects of empowerment that have emerged from the analysis: the enhancement of self-esteem and self-respect among queer producers, the heightened motivation observed among queer co-producers to initiate artisanal food projects, and the pursuit of a flourishing social life and a stronger connection to agriculture among queer co-producers.

GUA created an informal network for small-scale producers and consumers to sell their artisanal food production and food surplus. Ana and Antônia found in the collective financial arrangement of GUA an economic opportunity for their small cheese, bread and pastry production. Additionally, beyond the economic benefits, they highlighted two forms of professional and personal recognition from CSA members that contrasted with the discriminatory experiences in the local conventional agri-food system.

First, Ana celebrated the *collaboration with co-producers* that provided her with recognition and valorisation for her work and contributed to the viability of her artisanal production:

I am grateful for the CSA because it allows me to exist. It is a place where I can express myself, where I can be creative and where I am respected for the work I do. CSA members trust and appreciate my work. When selling outside the CSA, I am viewed as unprofessional, and my cheese production is viewed only as a hobby.

Ana herded a small batch of 15 goats and mentioned that rigid gender roles and intimate relationships in agriculture influenced who was deemed eligible to buy land in rural Alentejo. In Portugal, only 15.1% of farm managers are women, which is similar to the number in the Alentejo region, 13.4% (INE, 2021). Although these numbers do not concern land ownership, it shows that women are rarely in charge of Portuguese farms. Ana encountered several difficulties in leasing land to expand her goat herd. She experienced discrimination for being a woman seeking land– more specifically, for being a woman without a male partner:

I want to lease land for more space for the goats, and I can’t. That’s only because I’m a woman. Maybe if I were married to a man, my husband would be able to help me lease land. But I’m a woman, so they don’t trust that what I’m doing is serious. Agriculture and animals are a man’s job here. So, I am like a joke to them.

Concerning land ownership, Ana and Antônia commented that it was unusual for two women to buy farmland in rural Alentejo. For instance, several neighbours inquired about their relationship status when they first arrived, implicitly suggesting they were not entitled to be landowners: “there was much questioning. People wanted to know about us and wanted us to confirm that we were a couple.”

Second, GUA boosted Ana’s and Antônia’s *self-esteem and self-respect* as queers and food producers:

Since the CSA meetings happen at our place, and we are a couple, this is not a concern to anyone. I do not need to pretend we are not a couple, or people do not seem to be uncomfortable because we are a couple. This is already a big step. The CSA members treat us as a couple, not as friends. We are a couple; we are a family. They not only accept it but also respect it. This is *very* important for me. (Ana)

Ana and Antônia commented that people from their neighbouring farms called them “the Brazilian girls”. In this case, their gender and nationality obscured their occupation and intimate relationship. However, during the interviews and focus group with other queer and non-queer CSA members, participants referred to them by their names, occupation and intimate relationship, as exemplified in Miguel’s comment:

Before meeting Ana and Antônia, a goatherder that we both knew often spoke about them to me as “the Brazilian girls”. I wondered why he spoke about them as “the Brazilian girls”. […] After meeting them I realised they were a Brazilian couple producing food.

In effect, Ana and Antônia felt more discriminated against for being Brazilian women than for being queer in rural Alentejo: “We faced discrimination less for our queer identities and more as immigrant Brazilian women. The native Portuguese population in this region tends to stereotype Brazilian women as sex workers” (Antônia).They reported several cases in which they were mistakenly assumed to be sex workers by their neighbours.^11^ Although Brazilian sex workers worked at a brothel near their town, Ana and Antônia claimed that the comparison between Brazilian women and sex workers was linked to a cultural connotation of Brazilian women and migrants in Portugal^12^.

Furthermore, besides offering a market opportunity for queer small-scale producers, GUA encouraged queer co-producers to start and test their artisanal food production. For example, Miguel started an *empanadas* production after joining GUA and Maria, who was passionate about tofu found the motivation to start her artisanal production after realising that other CSA members were interested in the product. Moreover, the perception of the CSA as a supportive network, and not necessarily the experience in itself, might have been enough for queer co-producers to kick off their artisanal projects. Laura found inspiration from other queer project leaders in the GUA, which made her feel an *increased inherent motivation and ability* to start her seed-saving project:

The CSA is a space where small producers can start and try out and see if people like the product, get feedback, and so on. It’s not like you’re selling it at an anonymous supermarket. In that sense, it’s also empowering. And then, I guess, the queer side, for me, is empowering because I can see these examples led by other queer people.

GUA organised intermittent voluntary farm work to expand the partnership between producers and co-producers. Often, voluntary work was followed by convivial moments. Queer co-producers commented that agri-food and convivial activities allowed them to *pursue a flourishing social life and connection to agriculture* in rural Alentejo. Adilah participated in one-off volunteer farm work at different farms associated with the CSA. For Adilah, these were crucial moments to materialise her desired connection to agriculture and to actively participate in distinct phases of food production, from planting to harvesting: “I see the CSA farms as places I want to be close to. I like to go there and see how things are growing, what is growing, and how it has changed.” Similarly, Matteo explained that voluntary work for the GUA enabled him to strengthen social ties with other members of the collective while openly expressing his queerness:

I liked working with others, sharing the work, and connecting food with more social activities. Because the CSA is about nutrition, not only physical nutrition but about nurturing relationships. […] I don’t have many social connections in this region, so I don’t normally express my sexuality publicly. While in the CSA, the social connections are tighter, which is why I am there with my husband.

Although most queer members celebrated the combination of sociability and work in GUA, this empowerment experience also implied contradictory feelings in some cases. For co-producer Andreas, a 50-something-year-old gay cis-man, social interactions in GUA were intimate and deprived of anonymity which, in turn, made it hard to position himself in the group: “Being in this in-between private and public is hard for me. How do I talk to other people? As a private person? As a public person?” As a result, Andreas rarely attended GUA’s convivial moments and preferred baking the cakes his partner brought for potlucks.

In sum, GUA expanded and diversified the partnership between producers and co-producers. Financially, it created an informal network to commercialise and exchange artisanal food production and food excess. For queer producers, GUA offered economic opportunities along with recognition and valorisation of their informal and small-scale production that, in turn, were unappreciated in the local conventional agri-food system. For queer co-producers, GUA functioned as a supportive network that motivated them to kick off artisanal food production. Identity-wise, this partnership encouraged queer producers to pursue their farming careers and express their queerness simultaneously. Work-wise, GUA brought co-producers closer to the farmland and food production by combining voluntary work and convivial moments. While it enabled queer co-producers to pursue a flourishing social life and connection to agriculture in rural Alentejo, it also created contradictory experiences related to reputation and anonymity in the group.

#### Horizontal organisation: “It was amazing to encounter this group of people who shared this perspective with me and realise that many were queer living in the countryside!”

In this section, I introduce the types of empowerment experienced by queer CSA members in relation to GUA’s horizontal organisation. GUA created a democratic platform for collaborative decision-making that helped enhance self-confidence to shape decisions. Queer members felt empowered to establish reciprocal and collaborative relationships with other queer and cis-hetero members to build their envisioned agri-food system; however, this collective agency was limited to a privileged profile of queer rural people.

GUA offered its members a democratic platform to develop a community economy. The group employed several mechanisms inspired by sociocracy^13^ to foster participation and transparency in decision-making. For instance, every member could suggest contractual terms to fine-tune the responsibilities of co-producers and producers. Particularly in the case of horticultural production, producers and co-producers gathered before a harvest season (every six months) to assess the prior season’s pros and cons and collectively decide what to grow in the upcoming one. The story of co-producer Adilah illustrates the benefits provided by GUA’s horizontal organisation. Adilah viewed the harvest evaluation meetings as an opportunity to express her opinions and desires for the vegetable basket. Adilah explained that the participatory and collaborative decision-making features of the CSA heightened *her self-confidence to shape decisions* precisely because they released the burden of the responsibility to make individual decisions that affected the whole group:

There is clear communication and clear agreements. There’s co-participation, so I feel like I have a margin to change things I don’t like. It’s not a matter of, “Oops, I don’t like it, I’m leaving” or, “I like it, I stay”. As far as possible, I influence how the CSA system works. I can speak about my needs without fear. Sometimes they will be fulfilled, sometimes not. But at least I can give my feedback. There is an opening for us to decide together.

Although the evaluation gatherings were crucial moments for participants to embody the horizontal organisation envisioned by GUA, some queer members said that the meetings’ length and deliberations posed restrictions. For instance, for co-producer Laura, the evaluation meetings required articulation ability and time availability, which was not always compatible with members’ capacities and agendas.

Collaborative work between GUA producers and co-producers concerned convivial and agri-food activities while implicitly connecting queer people in rural Alentejo. In effect, several queer co-producers viewed the implicit queer inclusiveness of GUA as beneficial. On the one hand, it helped *establish reciprocal and collaborative relationships* with other queer and cis-hetero members to build their envisioned agri-food system. On the other hand, it helped avoid the stereotypes or stigma associated with queerness. For Miguel, the queer community in GUA was perceived as an aspect that enhanced, rather than pre-conditioned, his participation:

What attracted me to the GUA was the group of people in it, with whom I shared a common interest in food and how to treat the Earth and one another. That was more attractive than the fact that some were queer like me. However, it was amazing to encounter this group of people who shared this perspective with me and realise that many were queer living in the countryside!

Notably, it was only in the focus group that participants explicitly discussed their queer collective identity for the first time. Focus group participants mentioned they had never addressed inclusivity and protection strategies for queer members in GUA. Co-producer Laura sketched two possible future scenarios for dealing with their collective queer identity: either (i) keeping their queer identity implicit, which implied limited outreach to queer dwellers spread in the territory but avoided confrontation with heteronormative values reproduced by the broader local community, or (ii) making their queer identity explicit to allow queer dwellers to know that a local queer community existed but risking a backlash from conservative segments of the local community. In response to Laura, producer Ana referred to the second scenario as “raising the queer flag” and argued that it was undesirable in the case of GUA. For Ana, the queer flag was to be raised if the queer members felt threatened or attacked in or outside the CSA and needed protection. Similarly, producer Antônia argued that the CSA did not need to “raise the queer flag” to create a protective space against sexism or homophobia. In her view, this approach has helped create affinity and alliances with conservative cis-hetero neighbouring farmers who would not participate in queer circles in the absence of a common interest, which, in this case, was farming and food. Although the focus group conversation did not reach a consensus, participants seemed to agree with Antônia’s proposition that the CSA should aim to maintain their queerness implicitly and continue to create a safe space for queer peoples in the countryside, something that Antônia referred to as “working to keep the queer flag low”.

Furthermore, interviewees indicated that another core aspect of their collective identity and collaborative work was their privileged socio-economic and intellectual background, which some called “a bubble of privileged people in the countryside”. On the one hand, many members celebrated the safety and security created by this “bubble” effect. Miguel, for example, highlighted that the shared progressive values and worldviews among queer and hetero-cis members fostered collaborations through which queer people felt comfortable expressing their opinions, utilising their abilities, and pursuing their interests without fear of rejection or discrimination based on their gender and sexual identities.

On the other hand, some members expressed criticism regarding this socio-economic privilege. They raised concerns about the potential segregation of the community from the broader local population. Andreas commented, “I mean, the CSA is a bunch of privileged people. We are well-educated, usually have international experience, and have enough money to afford the prices of the CSA.[…] It is a community that is not representative of all the people in this region, of course”.

Another co-producer, Sophia, a 20-years-old cis-woman whose sexuality was undefined, echoed these concerns, sharing her experiences of encountering resistance when discussing the environmental concerns, principles of food sovereignty and autonomy she learned in GUA with friends, teachers and neighbours born and raised in the region. Similarly, Sophia commented that the enhanced sense of comfort to express queerness in GUA was less present when integrating other associations and cooperatives in the region, where sexual and gender diversity was less visible and obscured by the prevailing heteronormative culture. Both Sophia and Andreas recognised that the progressive lifestyles, worldviews, and conscious food production and consumption habits in GUA contributed to sexual and gender diversity and visibility in the collective, yet very distant from the reality of the broader local population. They considered this aspect of the initiative controversial and believed it had not been adequately acknowledged or addressed in internal meetings.

In sum, the GUA created a democratic platform to develop a community economy, which helped queer members enhance their self-confidence to shape decisions. Nonetheless, decision-making was lengthy and required substantial commitment from CSA members, which posed restrictions for participation. GUA foregrounded collaborations among members to construct an alternative local food system and implicitly created a queer-inclusive community. GUA opted not to raise the queer flag and keep their queer-inclusive collective identity implicit, which was also viewed as a strategy that helped create affinity and alliances with conservative cis-hetero neighbouring farmers. Interviewees noted the “bubble” effect resulting from the direct participation of privileged rural queer people in GUA, which provided a safe space for expression but also raised concerns about segregation and differences with the broader local population.

### Four types of queer empowerment

The findings revealed that queer members’ empowerment reflected various forms of power from within, power over oneself, power with and power to pursue one’s own flourishing through community relationships, producer–co-producer partnership and the horizontal organisation of GUA (Table 1). GUA fostered collective participation without specific focus on queer empowerment. Notably, the leadership of queer producers and recurrent gatherings at queer-owned farmland were crucial for expanding the empowerment potential of GUA to encompass gender and sexuality diversity, inclusivity and flourishing. Also, the contrasts between interviewees’ experiences in and outside GUA illuminated (c)overt heteropatriarchal discrimination in their interactions with local agri-food actors unrelated to the CSA. Queer cis-women, mainly Brazilian migrants, reported microaggressions and outright harassment while seeking farmland, buying food in local agri-food venues and integrating into the local rural community.

**Table 1:** Four types of empowerment identified in the case study

Type of empowerment	Characteristics	Empirical findings	
		CSA dimension	Queer empowerment
Power from within	Self-confidence, self-esteem and self-respect consistent with life-affirming force	Producer–co-producer partnership	Queer producers felt heightened self-esteem and self-respect for being queer and farmers
			Queer co-producers felt an increased ability and motivation to start artisanal food projects
Power over oneself	Mastering personal emancipation and the ability to decide one’s own life in resistance to oppression	Community relationships	Queer members experienced a heightened feeling of self-confidence that deactivated internalised oppression and naturally expressed their queerness
			Queer members took control over their queer selves when selling or purchasing food
Power with	Ability to act in concert to address issues and shared goals and undergo a liberatory process	Producer–co-producer partnership	Queer producers received recognition and valorisation from co-producers, and this partnership offered economic opportunities for artisanal production
		Horizontal organisation	Queer members established collaborative relationships to build their envisioned agri-food system while implicitly forming a queer community
Power to pursue one’s own flourishing	Self-entitlement and capacity to seek and choose one’s basic flourishing	Producer–co-producer partnership	Queer co-producers expanded their identities and explored thriving sexual expressions in the countryside
			Queer co-producers viewed the CSA as a means to pursue a flourishing social life and connection to agriculture in the countryside
Based on Allen (2021)			

Two crucial empowerment experiences contributed to the active involvement of queer people in GUA: *power with* emerging through collaborations to build an envisioned agri-food system and *power over oneself* related to a heightened sense of self-confidence that deactivated internalised oppression and enabled queer expressions. Two pertinent examples from this case illustrate how daily collaborative interactions imbued with self-confidence supported and reinforced, to different extents, other queer empowerment experiences within the collective. First, these interactions reinforced *power from within* as queer members felt self-confident to pursue farming careers, develop artisanal food projects and actively shape the functioning of the CSA system. Second, they influenced *power to pursue one’s own flourishing* as queer people actively pursued a connection to agriculture, a flourishing social life and an expansion of their sexual expression through their engagement in the collective despite the heteropatriarchal norms embedded in rural Alentejo.

Several contradictions and limitations of empowerment were identified in this case study. Queer empowerment in GUA was limited to a socio-economically and intellectually privileged group in rural Alentejo, whose values and class profile aligned with the nature and financial operations of the CSA scheme. Although the small number of participants and the highly valorised social and convivial moments enabled the creation and maintenance of personal connections in the CSA, this close-knit environment also dissolved anonymity in the group and restricted the participation of some queer members. Furthermore, regarding participation, the queer-inclusive community in GUA was created rather implicitly, and the initiative never discussed queer representation, inclusivity and protection, thus hampering the creation of internal agreements and measures for collective accountability of individual concerns. Moreover, participatory decision-making was a crucial democratic process to foster self-confidence and accountability over the organisation and implementation of CSA operations; however, it was exclusive to queer participants that wished and were available to participate in lengthy deliberations requiring articulation skills.

## Discussion: Three lessons from queer empowerment in CSA Guadiana

In this section, I draw three lessons from the empowerment stories of queer people in GUA. Each lesson discusses the main findings presented in the previous section “Four types of queer empowerment” and their implications for studies on relational agriculture within rural queer studies, including recommendations for future studies. To start, I offer new insights into the rural politics of recognition based on interviewees’ self-confidence in expressing queerness in GUA. Then, I discuss how GUA’s producer and co-producer partnership casts reciprocity as a relevant tactic for queer community action in the countryside. Last, I expand the notion of rural queer visibility based on the case of queer producers’ leadership in GUA.

### Recognition: Self-confidence to express queerness in a *selective* rural community

The rural politics of recognition refers to the tactics of queer rural people to assert their sameness and ensure acceptance in rural communities (Gray 2009; Hoffelmeyer 2021). However, the rural politics of recognition embodied by queer members within GUA offer a different reading of the maxim “We are just like everyone else” (Gray 2009, p. 38): queer members asserted their sameness in the collective as “just another queer person” and gave visibility to their queerness with confidence and enjoyment, instead of downplaying gender and sexual identity differences to ensure acceptance. Queer people in GUA performed what Velicu (2023) calls the disidentification of the peasant and queer categories. To different extents, they lacked recognition and valorisation as artisanal food producers and queers in rural Alentejo’s conventional agri-food system, which was rooted in heteropatriarchal values and oriented towards industrialised agriculture. Whereas in the CSA, queer people discarded these categories and their oppressive connotations to fulfil their desires and engage with artisanal food production while simultaneously feeling self-confident in expressing queerness.

One limitation of the empowerment capacity of GUA concerns the profile of the members. From an intersectional standpoint, GUA enabled a gender and sexually underrepresented group to flourish in a rural area. However, the same group was mainly neo-rural and enjoyed a degree of socio-economic and intellectual privilege that enabled them to comply with the principles and prices reproduced in the CSA that, in turn, were not representative of the worldviews and practices of most of the broader local population. This finding aligns with previous claims from gender studies on CSA that CSA initiatives are often composed of middle-class, well-educated and white participants (Cone and Myhre 2000; Delind and Ferguson 1999; Jarosz 2011). A deeper examination of which strategies could be used by CSA to ensure further inclusivity and enable rural queer dwellers of different socio-economic backgrounds to be empowered would supplement the understanding of queer empowerment in CSA–more specifically, how the CSA could re-organise itself internally in ways that queer empowerment goes hand-in-hand with socio-economic diversity from a class perspective. Additionally, it is crucial to explore strategies that authorities, organisations and CSA networks can employ to promote and disseminate this agri-food model in ways that facilitate gender and sexual diversity across social classes. Well-known strategies to promote CSA that could be further explored to include gender, sexuality and class dimensions include community land trusts that provide low-cost secure tenure rights (Paul 2019), public procurement aligned with the production capacities of CSA initiatives (Bonfert 2022), and supportive legal and tax systems for small scale-producers(Kapała 2020).

### Community action: *Reciprocal* relationships among diverse queer agri-food actors

The producer–co-producer partnership performed in GUA casts new light on the potential of community action for rural queer empowerment. Members committed to meeting weekly during harvest season and actively shaped the CSA system. Recurrent gatherings showcase a viable strategy for community action that provides an alternative to participation restrictions reported in queer farmers’ networks because of the geographic dispersion of members and few gatherings (Hoffelmeyer 2021). Additionally, while queer farmers’ networks are mainly composed of queer farmers (Leslie 2019; Wypler 2019), the partnership dimension of GUA reached a wider queer population involved in the local agri-food system of rural Alentejo also including artisanal food producers and consumers. In doing so, GUA created a communitarian and an extended-responsibility approach to farming and food provisioning beyond the traditional nuclear family farm model: its economic arrangement open up possibilities for queer farmers’ businesses and provided recognition and valorisation for their artisanal production and queer identities otherwise discriminated against by the local conventional agri-food system.

While this study represents an initial step in bringing the literature of rural queerness into conversation with the scholarship on CSA to examine the experiences of queer people in agri-food community action, the use of a single case study limits the generalisability of findings. The case of GUA enabled a deeper understanding of the nuances and complexities of queer empowerment in a CSA initiative. Yet, to grasp more comprehensively the potential of the CSA model in empowering rural queer people, future research could benefit from engaging with comparative analyses exploring other dimensions of the CSA model beyond those considered for this study. For instance, diverse approaches to the producer—co-producer partnership, such as instrumental, functional or collaborative (Feagan and Henderson, 2009), and distinct structures and aims of organisational formats, including farmers-, consumers- and cooperative-led CSA (Degens and Lapschieß 2023; Gorman 2018; Piccoli et al. 2021) may enable different levels of reciprocal relationships among queer producers and co-producers. Accordingly, I envision future research that refines and provides more nuance to the potential of diverse CSA models in empowering rural queer people.

### Visibility: *Quiet* queer representativity in and beyond GUA

In contrast to visible forms of queer community action against heteropatriarchy in rural agri-food systems (Leslie 2019; Wypler 2019), the case of GUA foregrounds a “quiet form of activism” (Delind and Ferguson 1999): a CSA that does not “raise the queer flag” but rather “works to keep the queer flag low” and implicitly creates a safe space for rural queer people to get involved with agriculture. Quiet, in this sense, is not related to a constrained queer agency such as “hiding in the closet”. Instead, it refers to forms of emancipation from heteropatriarchal discrimination, and queer representativity lived through everyday relationships in the CSA when creating and negotiating the approach to food, the environment and people. Although quiet activism falls short in catalysing fundamental transformation in agri-food systems (Delind and Ferguson 1999), in the case of GUA, it helped create affinity between queer and cis-hetero members and between them and cis-hetero farming neighbours.

Remarkably, the leadership of queer producers in GUA influenced the type of everyday relationships in the collective and was essential to tailoring the empowering potential of GUA to queer participants. Similar to previous claims that CSA producers highly influence the level and degree of members’ involvement in CSA initiatives (Raj et al. 2023), queer producers’ self-assertive attitude towards their queerness and intimate relationship was evident during GUA gatherings at their farmland and influenced the participation of other queer members. Queer co-producers discarded the need to protect and declare their queer identity and felt self-confident in pursuing their desired connection to agriculture, enjoying a flourishing social life and, in some cases, further exploring their rural identity and sexual expressions. While this study only analysed the benefits and limitations of quiet queer activism in GUA, future research on rural community action against heteropatriarchy in the pursuit of sustainable agri-food systems may benefit from comparing the advantages and disadvantages of visible and quiet forms of activism across different agri-food grassroots initiatives.

## Conclusion

In this study, I analysed the experiences of 12 queer members of a CSA located in rural Alentejo, South Portugal, and asked whether and how they felt empowered to become thriving and active members of the collective. Drawing on the notion of “relational agriculture” (Leslie et al. 2019), I approached queer empowerment through the intersection of gender, sexuality and agriculture. From the standpoint of this gender and sexually underrepresented group in rural Alentejo, I analysed four types of empowerment, including their contradictions and limitations, influenced by the community relationships, producer–co-producer partnership and horizontal organisation dimensions of CSA.

The results revealed that these three dimensions of GUA were not tailored to queer empowerment; yet, queer producers’ leadership in the CSA and the recurrent organisation of CSA gatherings in their queer-owned farmland were crucial for expanding the empowering potential of GUA also to include gender and sexuality diversity, inclusivity and flourishing. Mainly, queer members found in GUA a social, physical and cultural space to safely develop collaborative agri-food operations with other queer and cis-hetero people while feeling self-confident in expressing their queerness. These experiences contrasted with other interactions with local agri-food actors unrelated to the CSA in which queer people encountered (c)overt forms of heteropatriarchal discrimination. Despite these emancipatory achievements, queer empowerment in GUA was exclusive to a socio-economically and intellectually privileged group and restricted by the lack of internal debates on gender and sexuality, as well as by the time and articulating abilities needed to participate in internal decision-making processes.

This paper contributes to debates on relational agriculture within rural queer literature by addressing the call for studies about the struggles and achievements of gender and sexually underrepresented groups in CSA (Leslie 2017). It offers new insights into several manifestations of empowerment, including its contradictory outcomes and limitations experienced by queer farmers, artisanal food producers and consumers in a rural CSA (Table 1). Based on these findings, this paper draws three lessons relevant for the further theorisation of relational agriculture: (i) self-confidence to perform queerness in rural CSA may be restricted to a selective rural community, (ii) producer and co-producer partnership in CSA may enable reciprocal queer empowerment and (iii) queer producers’ leadership in CSA may quietly represent gender and sexual diversity in rural communities.

## Abbreviations

***CSA***: Community-supported agriculture
***GUA***: Community-supported agriculture Guadiana

## Footnotes

1 The term ‘queer’ is highly contested and central to distinct yet interconnected debates. For instance, queer identity politics focuses on the experiences, identities and lived realities of LGBTQIA + individuals (Butler 1990). Queer political theory explores the intersection of queer issues with power, governance, law and public policy (Preciado 2018). Queer ecology examines the intersection of queer issues with ecological concerns, environmental justice, and biology (Sandilands 2002). In this study, ‘queer’ and ‘queerness’ are used in alignment with research on queer identity politics in rural and agriculture communities (Leslie 2017; Wypler 2019; Hoffelmeyer 2021) and refer to individuals who are non-heterosexual and non-cisgender.

2 GUA adopted the term “co-producer” as an alternative to the term “consumer”. Generally, the new term intended to spur active participation and shared accountability over the economic viability and labour for agri-food production, in contrast to the passive role of consumers performed in conventional market transactions. Nonetheless, this intended behaviour and mindset shift remained a challenge, as co-producers had a significantly smaller work share than farm owners and employees, and their involvement in work tasks was optional.

3 The profile of rural queer people can be highly diverse and entails gender and sexuality experiences in relation to other aspects of the social life in the countryside (e.g., class, family constellation, race). A well-known distinction in the profile of rural queer peoples in rural queer debates is the one between queers born-and-raised in the countryside and neo-rural queers (Bell and Valentine 1995). In this study, I refer to “rural queer peoples” generally as a group of rural queer dwellers, either born-and-raised, or not, in the countryside.

4 Heteropatriarchal discrimination “is a set of racialized, gendered, and sexualized power relations that privileges those who are white, cisgender, men, and/or heterosexual and limits human resources for those who do not and cannot fit these boxes” (Wypler 2019, p. 984). Heteropatriarchal logics may be incorporated into one’s subjectivity and inform discriminatory acts across and beyond all genders.

5 I use the term “cis-hetero” as an abbreviation of the terms “cisgender” and “heterosexual” combined.

6 The literature used in this study regarding rural queerness and gender relations in CSA is primarily focused on the Western and Global North contexts, both in terms of empirical research and onto-epistemological perspectives. Consequently, the scope and nature of the analysis in the study are inherently influenced by this limited context. However, it is important to acknowledge that experiences of gender and sexuality in non-Western and non-Northern contexts, particularly in rural areas and within CSA, prompt crucial questions about empowerment and the generation of knowledge that extends beyond the Western scientific paradigm.

8 The term “neo-rural” is closely associated with the concept of “new rurality” (Marsden, 1998; Wright and Annes 2014). New rurality highlights the evolving and adapting character of rural population demographics, in which neo-rurals engage with non-traditional activities in rural areas and develop projects such as agro-tourism and alternative agriculture. In the context of Portugal (Esteves 2017; (Novikova 2021)), neo-rurals are generally middle-class young people who migrate from urban areas seeking lifestyle changes and proximity to nature.

9 Participants indicated the ethnicity “Mediterranean” when referring to either the European Mediterranean population in the south of the continent or the African Mediterranean population in the north of the continent that are deeply marked by migratory flows historically characterising the Mediterranean region.

10 The interviewee described a creative gender as a gender that is not fixed nor closed to a single category, but instead they understand gender as a category to be continuously put into question and always in the making.

11 In this case, Ana and Antônia referred to the negative connotations of sex workers. However, I acknowledge that this connotation is a social construct imbued with discrimination and obscures the dignity and working rights of sex workers.

12 In this case, heteropatriarchy intersects with xenophobia and informed discrimination towards queer Brazilian farmers, the largest immigrant population in rural Alentejo (INE, 2022). Historically, Brazilian women have been exposed to colonial and gender discrimination in Portugal (Gomes 2018).

13 Sociocracy is a method of self-governance that features decision-making mechanisms based on consent and participatory tools for co-creating and implementing proposals for collective modes of organisation (for a case of sociocracy in CSA, see Cristiano et al., 2020).
